# A novel approach for contactless heart rate monitoring from pet facial videos

**DOI:** 10.3389/fvets.2024.1495109

**Published:** 2024-12-02

**Authors:** Renjie Hu, Yu Gao, Guoying Peng, Hongyu Yang, Jiajin Zhang

**Affiliations:** ^1^College of Big Data, Yunnan Agricultural University, Kunming, China; ^2^College of Mechanical and Electrical Engineering, Yunnan Agricultural University, Kunming, China

**Keywords:** contactless monitoring, heart rate, pet, welfare monitoring, iPPG, animal health

## Abstract

**Introduction:**

Monitoring the heart rate (HR) of pets is challenging when contact with a conscious pet is inconvenient, difficult, injurious, distressing, or dangerous for veterinarians or pet owners. However, few established, simple, and non-invasive techniques for HR measurement in pets exist.

**Methods:**

To address this gap, we propose a novel, contactless approach for HR monitoring in pet dogs and cats, utilizing facial videos and imaging photoplethysmography (iPPG). This method involves recording a video of the pet’s face and extracting the iPPG signal from the video data, offering a simple, non-invasive, and stress-free alternative to conventional HR monitoring techniques. We validated the accuracy of the proposed method by comparing it to electrocardiogram (ECG) recordings in a controlled laboratory setting.

**Results:**

Experimental results indicated that the average absolute errors between the reference ECG monitor and iPPG estimates were 2.94 beats per minute (BPM) for dogs and 3.33 BPM for cats under natural light, and 2.94 BPM for dogs and 2.33 BPM for cats under artificial light. These findings confirm the reliability and accuracy of our iPPG-based method for HR measurement in pets.

**Discussion:**

This approach can be applied to resting animals for real-time monitoring of their health and welfare status, which is of significant interest to both veterinarians and families seeking to improve care for their pets.

## 1 Introduction

Cats and dogs, the most popular pets in society, are frequently regarded as family members. Therefore, their health has received widespread attention in recent years. Considering that pets are unable to communicate and inform their owners when they are ill, monitoring their heart rate (HR) becomes extremely useful in detecting diseases and observing their behavior and responses to treatment (1, 2).

A significant increase or decrease in the HR may indicate severe illnesses such as dehydration, heart disease, fever, or shock. Furthermore, the HR is commonly used as an emotion-related physiological indicator for assessing the mental states of cats and dogs, such as anxiety and depression (3–6).

Currently, contact sensors are widely used for obtaining the HR of pets. However, this method frequently requires invasive preparation procedures and causes significant distress in animals (7). Thus, pets are occasionally anesthetized or placed under strict restraint to prevent movement that could disturb the measurement setup (8, 9). Traditionally, the most common contact-sensing tool for measuring the HR of a pet is the electrocardiogram (ECG), which is practices frequently adopted for HR waveforms. It requires stable electrical contact with the skin electrode during measurement for which hair may have to be removed; in some cases, even anesthesia may be necessary (10–12). A collar is another widely-used method for measuring HR via sensors that come into contact with the body of the animal (13, 14). However, the collar must be extremely tight around the neck of the pet if the sensor is to capture such signals, impairing the animal’s normal behavior and comfort.

Compared with contact sensors, contactless HR detection does not require sensors attached to the target body, contributing to improved target comfort and preventing changes in the physiological parameters of contact-sensitive pets caused by touch. Owing to their advantages, non-contact sensors have attracted considerable research attention. Photoplethysmography (PPG) is a widely-used optical technology used for HR monitoring (15–17). However, they cannot be used to monitor pet vital signs because they have a short detection range and are limited by the condition of the body surface of the animal. Similarly, hair covering the body surface also renders camera- or video-based approaches complex, limiting their application to animals (18, 19). Recently, radar, a contactless vital sign monitoring method, has received extensive interest and has been applied to various scenarios (20–22). Ultra-wideband (UWB) radar has been used to measure vital signs in dogs and cats (6). Suzuki et al. (23) proposed a respiratory monitoring system based on a microwave radar antenna operating at a frequency of 10 GHz to measure the breathing rate of a Japanese black bear during hibernation without any physical contact. A millimeter-wave radar was used to measure the vital signs of rats and rabbits (24). To be sufficiently sensitive to the vital signs of small animals, they raised the carrier frequency to the millimeter-wave level, which not only increased the system cost but also reduced the operational distance. However, the short detection range restricted its applicability in monitoring the vital signs of pets at home, and the sensor used in their study was limited to short distances within the electrical field, resulting in additional costs for modifications to the environment.

Thermal cameras have also been extensively adopted in animal research (25–28) because they are suitable for long-term monitoring in dark environments. However, the limitations of such measurements include the difficulty in extracting the signal with a partially occluded region of interest (ROI), ambient environmental thermal noise, high cost, and comparatively short distances owing to the low resolution, optics, size, and cost that are all connected to thermal imaging physics, and lack of a consumer market for the devices (29).

Considering the market demand, digital visible-light cameras may be a better choice for animal surveillance, as they offer at least three visible channels with high levels of resolution, intensity (bits per pixel), spatial (pixels per degree), and temporal (frames per second) capacity. Additionally, recording videos in a variety of settings is enabled by the flexibility of the visible-light optical design, which provides panoramic, microscopic, and telescopic solutions in seamlessly integrated commercial product families (30). Imaging photoplethysmography (iPPG) has been proposed (31, 32) as a remote and non-contact alternative to conventional PPG in humans. An iPPG is acquired using a video camera instead of a photodetector under dedicated or ambient light (31, 33–35). Videos are usually recorded from facial regions (36). Recently, Unakafov et al. (37) established a non-contact pulse-monitoring system to extract iPPG signals from red, green, and blue (RGB) facial videos of rhesus monkeys. Pilot studies demonstrated the possibility of extracting iPPG from anesthetized animals, particularly pigs (38, 39).

In the current pilot study, a new, non-contact, non-invasive, and cost-effective monitoring system based on iPPG was explored to extract the HR at different distances and lights using motion on the face of the pet. Continuous wavelet transformation (CWT)-based analyses were then performed on the extracted iPPG video signals. They were revealed to be motion tolerant on poor-quality video data (40, 41). Concurrently, comparison of the RGB three-layer color signals suggested that the red-layer signal was more suitable for the analysis of the HR signal of the pet and that selecting different signal channels according to different situations was more appropriate. Considering that iPPG enables easy and noninvasive estimation of pulse rate, it can be useful for pet studies. We minimized potential annoyance by using a non-invasive, contactless iPPG method, training animals to remain still with positive reinforcement, and conducting measurements in familiar environments, thus ensuring minimal stress and discomfort. Overall, this method can be generalized as a tool for tracking the HR of pets for etiological, behavioral, or welfare purposes.

## 2 Materials and methods

### 2.1 Animals and animal care

This pilot study included six dogs (two Chinese indigenous dogs, two Golden Retrievers, a Siberian husky, and a Border Collie) and five cats (two American Shorthair cats, two British Shorthair cats, and a Chinese indigenous cat). The patients were aged 3 months–9 years old. Details are presented in Table 1. The animals were acquired from Kunming Xinyi Pet Hospital (Kunming, Yunnan, China). The pets engaged in the current study had previously participated as subjects in ECG monitoring experiments, boasting a wealth of experience in this experimental setting.

**Table 1:** Specimen information.

Subject	Animal	Sex	Age	Weight (kg)	Species
D1	Dog	Male	1 year	7	Chinese indigenous dog
D2	Dog	Male	9 years	30	Golden retriever
D3	Dog	Female	3 months	3	Chinese indigenous dog
D4	Dog	Female	1 year	25	Golden Retriever
D5	Dog	Female	3 months	5	Border Collie
D6	Dog	Female	3 months	5	Siberian Husky
C1	Cat	Male	3 years	4	American Shorthair cat
C2	Cat	Male	7 months	3	British Shorthair cat
C3	Cat	Female	3 years	5	American Shorthair cat
C4	Cat	Female	4 years	4	British Shorthair cat
C5	Cat	Female	4 years	7	Chinese indigenous cat

This research was based only on filming, so that the routines of the animals were not disrupted. None of the animals had known health problems during the filming. For the current trial, we trained pets to perform a lie-down behavioral task for approximately 30 s and return on command. Each pet’s facial video was recorded at distances of 30 cm, 60 cm, and 90 cm under both natural and artificial lighting conditions. The duration of each video clip was 30 to 50 s, with a total recording time of approximately 5 to 7 min per pet to ensure sufficient data for heart rate estimation. The pets were individually moved from their home cages to the testing laboratory and calmly lie on the familiar reclining chair after receiving training with positive reinforcement.

All animals were handled in strict accordance with good animal practices as defined by the relevant national and local animal welfare bodies. All experiments were performed in accordance with relevant guidelines and regulations and authors complied with the ARRIVE guidelines.

### 2.2 Data acquisition and experimental setup

The videos were recorded using HD video cameras (iPhone 11) on October 14, 2022. Within the experimental plots, videos of the animal faces were recorded for each animal. Videos of each animal was recorded for 30–50 s at a frame rate of 30 fps, with frames measuring 1,280 pixels in width and 720 pixels in height, saved in MP4 on a laptop.

Fluorescent lamps mounted on the ceiling or walls of the room were used for illumination. The recorded video was compared to natural and artificial light. All videos were recorded under ambient (non-dedicated) light. Different illumination conditions were adopted to enable our video-based approach to cope with this variability because setting a particular illumination is often impossible in everyday situations.

**Figure 1:**
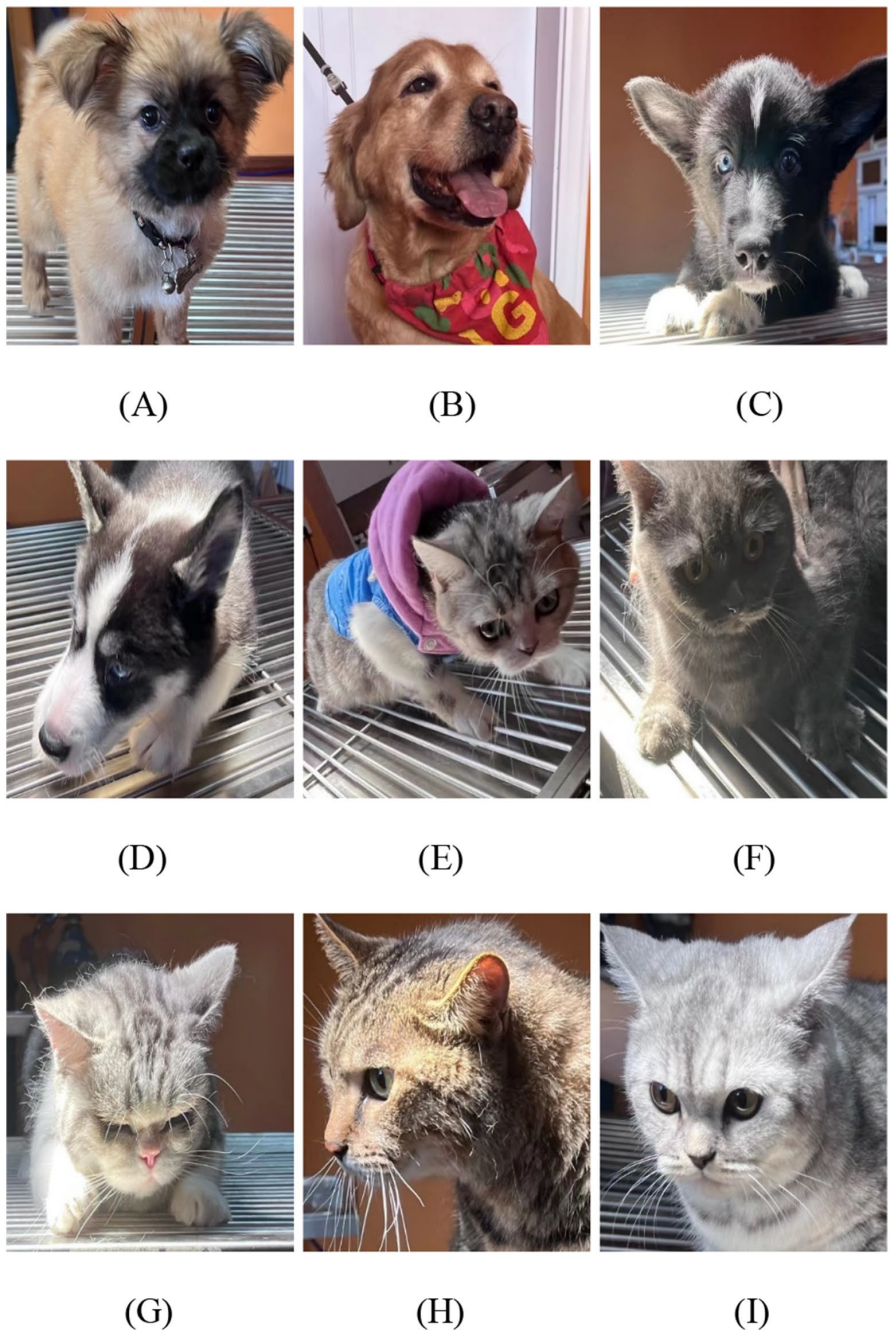
Data collection from animals. **(A)** Chinese indigenous dog. **(B)** Golden Retriever. **(C)** Border Collie. **(D)** Siberian Husky. **(E)** American Shorthair cat. **(F)** British Shorthair cat. **(G)** American Shorthair cat. **(H)** Chinese indigenous cat. **(I)** British Shorthair cat.

To cope with the variability in distances, distances of 30, 60, and 90 cm between the camera and the face of the animal were set for comparison. Figure 1 shows a single frame of the facial video data collected from the animals.

During the experiments, the pets were extensively trained with positive reinforcement, transported individually from their home cages to the testing laboratory, and lie on a familiar reclining chair. The positive reinforcement training helped ensure that the pets remained still on the reclining chair during the monitoring process.

Each animal underwent habituation prior to beginning data collection for this study to get accustomed to the chairing processes. This enabled video recordings to be taken while the animals worked on tasks in the ECG. Synchronized video recordings and ECG (3303 B, 3 Ray, China) measurements of the pets were conducted to verify the video-based measurements of the HR of dogs and cats.

**Figure 2:**
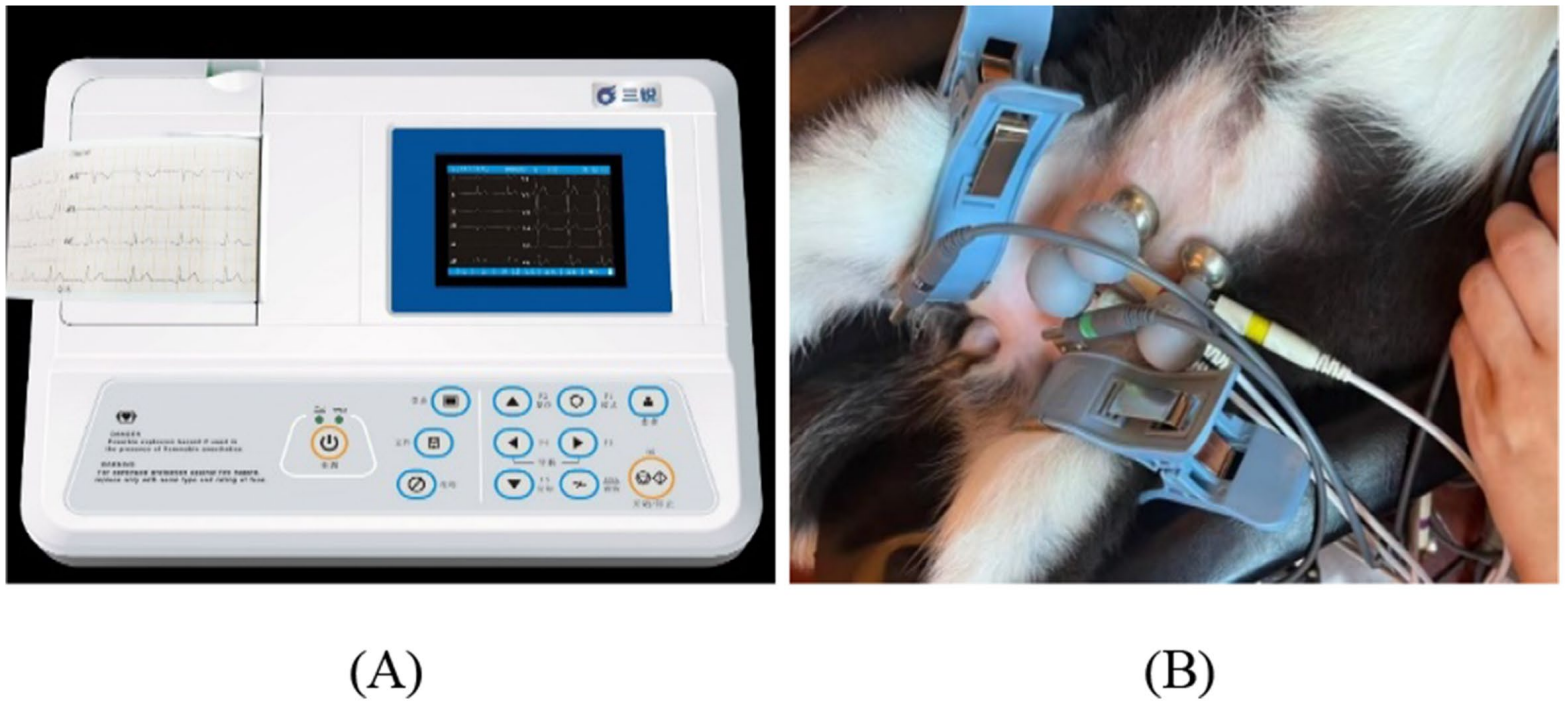
**(A)** Reference ECG module (3303 B, 3 Ray). **(B)** Experimental scene for dogs.

Figure 2 shows the experimental setup. The ECG monitor measures HR data during behavior. Spectral analysis was performed using the video and the data that the ECG recorded were compared with that from the spectral analysis.

### 2.3 System framework and data analysis

#### 2.3.1 System framework

**Figure 3:**
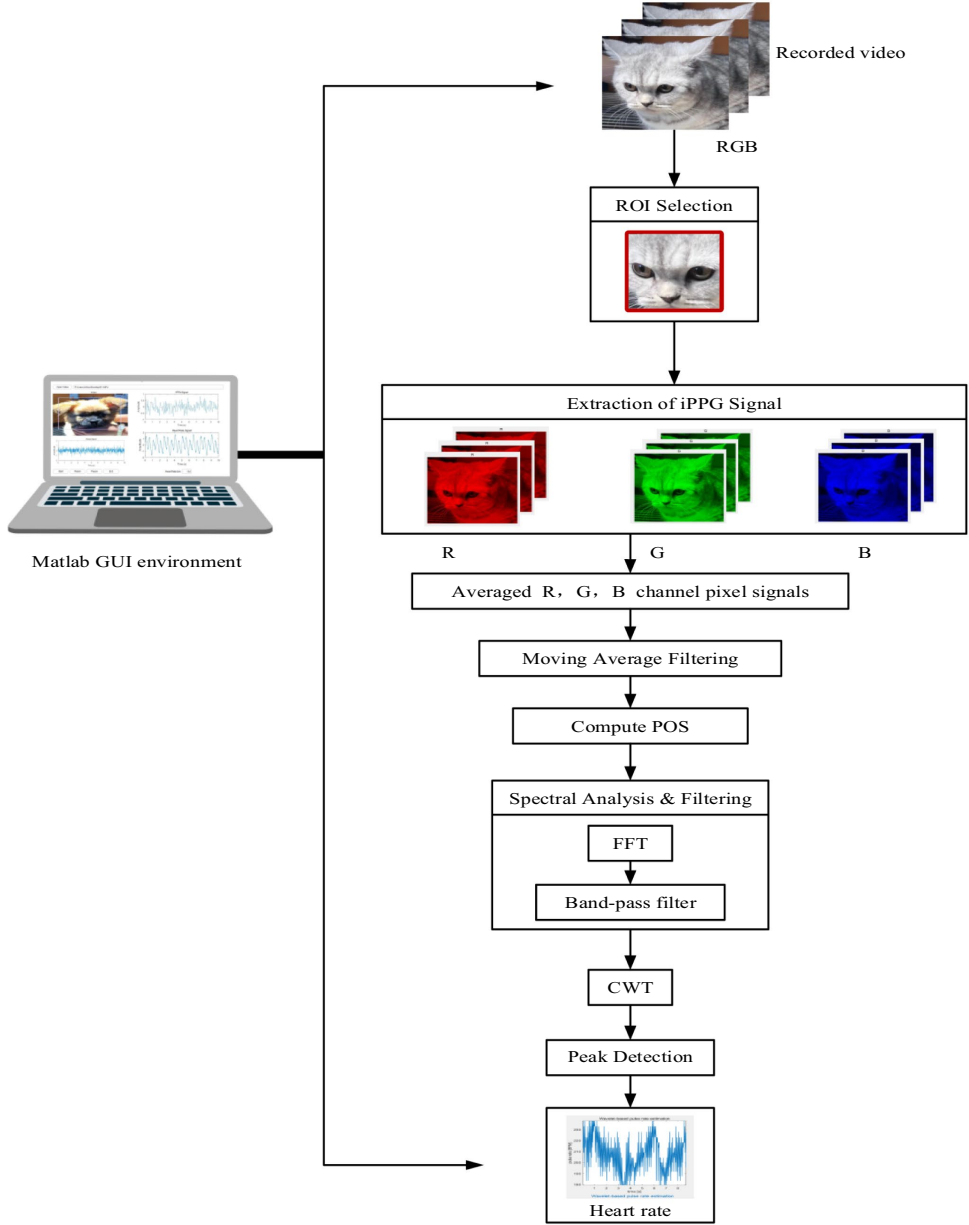
Schematic of the process by which contactless video data is obtained using an HD video camera (iPhone 11) to extract the HR of pets.

A schematic of the pilot system used to extract the HR of pets from video data is shown in Figure 3. This image outlines a system for extracting and analyzing iPPG signals. It starts with video recording and region-of-interest (ROI) selection, followed by the extraction and averaging of RGB channel signals to generate the iPPG signal.

The signal is then filtered, analyzed using plane-orthogonal-to-skin (POS) and fast Fourier transform (FFT), and processed with continuous wavelet transform (CWT) for feature extraction. Peak detection is performed to calculate heart rate, which is displayed in a MATLAB graphical user interface (GUI) environment. Facial videos of dogs and cats were processed to compute iPPG signals.

Video processing, ROI selection, and color signal computation were implemented in C++ using OpenCV,^1^ which is a widely used open-source computer vision library. iPPG extraction and processing, along with HR estimation, were performed using MATLAB 2016a,^2^ a powerful numerical computing environment.

#### 2.3.2 Region of interest selection

**Figure 4:**
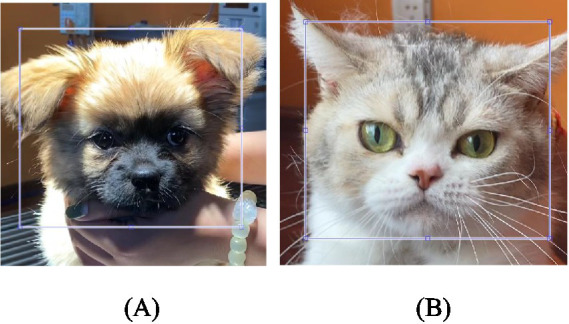
Regions of interest used for iPPG signal extraction from the **(A)** dog and **(B)** cat videos.

As shown in Figures 4A,B a rectangular boundary for the ROI was manually selected for the first video frame. This boundary remained the same throughout the video because no prominent motion was expected from head-stabilized dogs and cats. The regions with the best iPPG extraction for most sessions were the cheeks. The values of the color channels over the ROI were averaged to reduce spatially uncorrelated noise and enhance the pulsatile signal.

#### 2.3.3 Extraction of iPPG signal

**Figure 5:**
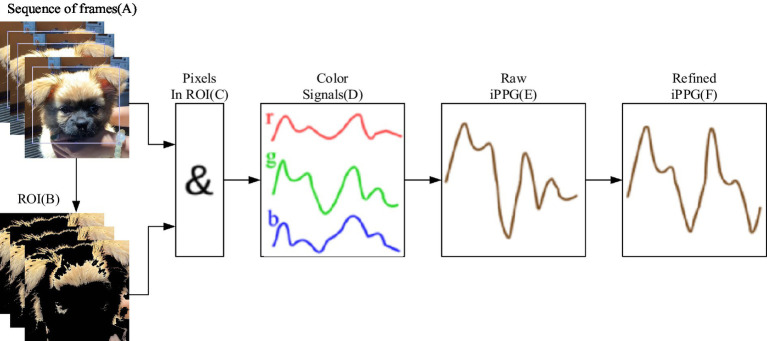
Flowchart of iPPG signal extraction.

Particularly, the selection of pixels that might contain pulse-related information is described in the extraction section (37), and the region of interest, computation, and processing of iPPG is refined in the extraction section and processing of iPPG. Moreover, to enhance the quality of the iPPG signal, the pixels containing the maximal amount of pulsatile information were selected, as shown in Figure 5. (A) A region of interest (ROI) is defined from a sequence of RGB frames. (B) For each frame, ROI pixels containing pulse-related information (shown in white) are selected. (C) For these pixels, across-pixel averages of unit-free non-calibrated values for red, green, and blue color channels (D) are computed. (E) iPPG signal is computed as a combination of three-color signals and then (F) refined using several filters.

#### 2.3.4 Processing of iPPG signal

The novelty of the proposed method is that it uses a plane orthogonal to skin tone in a temporally normalized RGB space for pulse extraction. This is named “plane-orthogonal-to-skin” (POS), which is also a unique character distinguishing it from prior work. Its algorithm was kept as simple as possible to highlight the fundamental/independent performance of the POS, although the commonly used band-pass filtering was not adopted. The bare core algorithm of POS is described in Algorithm 1 and can be implemented in a few lines of MATLAB code (35, 42).

##### ALGORITHM 1 Plane-orthogonal-to-skin (POS)

**Table:** 

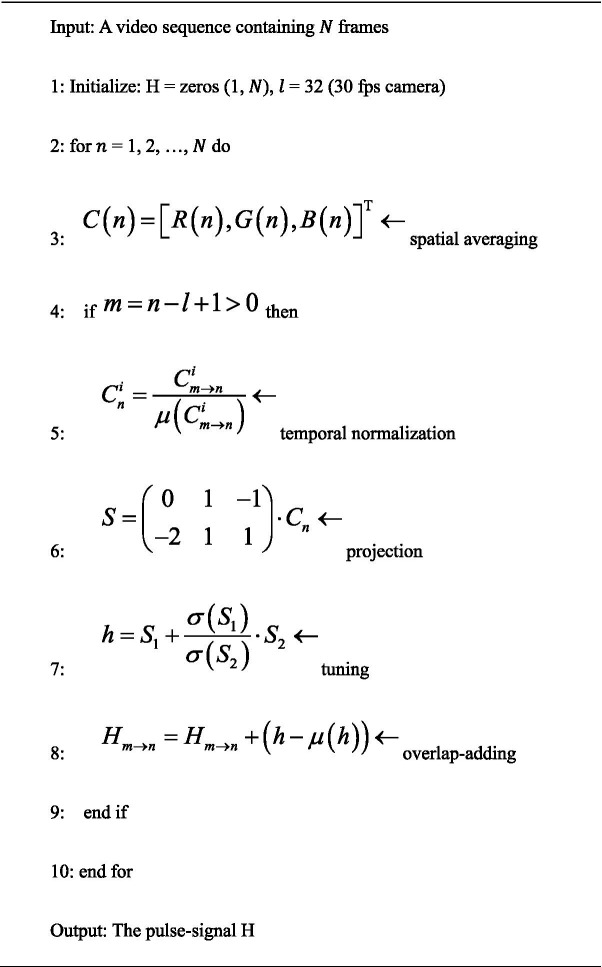

Thus, the task of extracting the pulse-signal from the observed RGB signals can be translated into defining a projection-system to decompose $C\left(t\right)$ . We project a temporally normalized RGB signal Cn , measured from the skin in a video.

Over a wide range of lighting spectra and commonly used camera sensitivities, the R-channel has the largest pulsatile amplitude, followed by the G-channel and B-channel, respectively. We project $Cn\left(t\right)$ onto the plane orthogonal to 1, which is expressed as $Sn\left(t\right)$ , and obtain $S_{1}$ , $S_{2}$ . We leave the task of finding an exact projection direction to the alpha-tuning, which can be expressed as h . Assuming that $h\left(t\right)$ is estimated from short video intervals in a sliding window (with length l ), we can derive a long-term pulse-signal H by overlap-adding the partial segments $h\left(t\right)$ (after making them zero-mean).

The implementation of POS strictly follows Algorithm 1 presented in this paper. The sliding window length of POS is defined as l = 32 given a 30-fps camera, which measures cardiac activities in 1.6 s, i.e., it can capture at least one cardiac cycle of the measured signal in a broad heart rate range [40, 240] beat per minute. The parameters in the benchmarked methods are set according to the original papers.

For fair comparison, all parameters remained identical when processing different videos. For each frame, all outlier pixels that differed significantly from other pixels in the ROI were excluded. This step eliminates the pixels corrupted by artifacts. Thus, pixel $\left(i,j\right)$ in the k -th frame are excluded if the value of any color channel $c_{k}^{i,j}$ does not satisfy the inequality (30) by the Equation (1):


(1) $$m_{k}-1.5\sigma_{k}<c_{k}^{i,j}<m_{k}+1.5\sigma_{k}$$

where $m_{k}$ and $\sigma_{k}$ denote the mean and standard deviation of the color channel c for the pixels included in the ROI of the k -th frame, respectively.

iPPG signals were extracted and processed. Color signals $r_{k}^{\prime }$ , $g_{k}^{\prime }$ , and $b_{k}^{\prime }$ were computed as averages for each color channel over the ROI obtained by refining the cheek regions for every frame k . Prior to iPPG signal extraction, each color signal $c_{k}^{\prime }$ was centered and scaled to make it independent of the brightness level and spectrum of the light source. Equation (2) is the standard procedure for iPPG analysis.


(2) $$c_{k}=\frac{c_{k}^{\prime }-m_{k,M}}{m_{k,M}}$$

where $m_{k,M}$ indicates an M -point running from Equation (3):


(3) $$m_{k,M}=\frac{1}{M}\overset{k}{\underset{l=k-M+1}{\sum }}C_{l}^{\prime }$$

For k<M we use $m_{1,M}$ . We followed (35) in taking M corresponding to 1 s.

**Figure 6:**
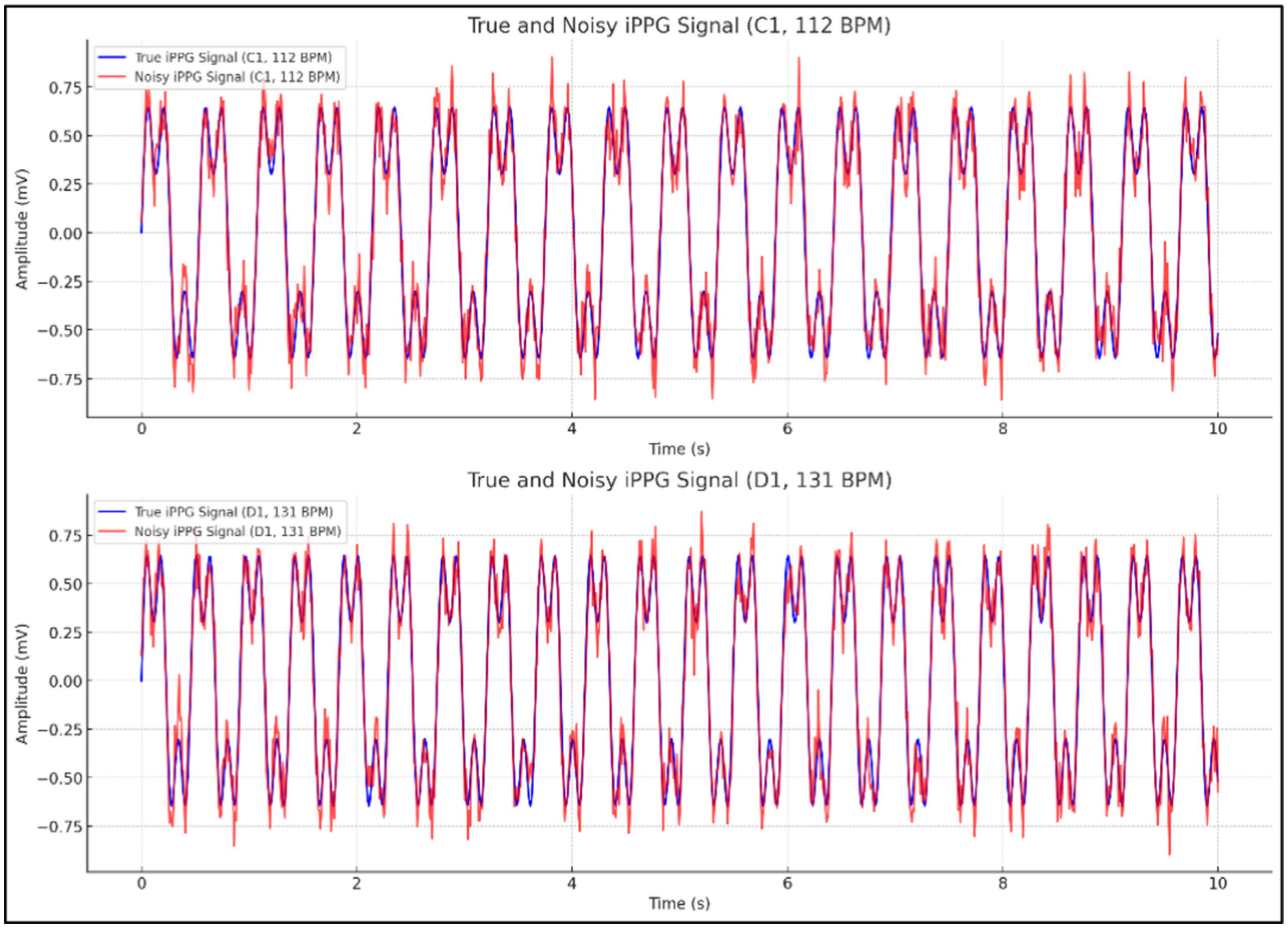
Comparison of true and noisy iPPG signals at varying heart rates.

As illustrated in Figure 6, the image displays two comparisons of true and noisy iPPG signals, one at a heart rate of 112 BPM (C1) and the other at 131 BPM (D1), highlighting how noise affects the accuracy of the signals under different conditions.

iPPG-based HR estimates were compared with reference estimates using a contact ECG monitor to assess the quality of the HR estimation. Additionally, percentages with estimation errors within a certain range are presented to facilitate the interpretation of the results.

#### 2.3.5 Signal analysis and selection of RGB component

The second phase of processing involved the manual identification of regions of interests (ROIs) within the facial area where the HR signal was subsequently analyzed using MATLAB’s inbuilt command “Gin put.” The ROIs are outlined as rectangles.

**Figure 7:**
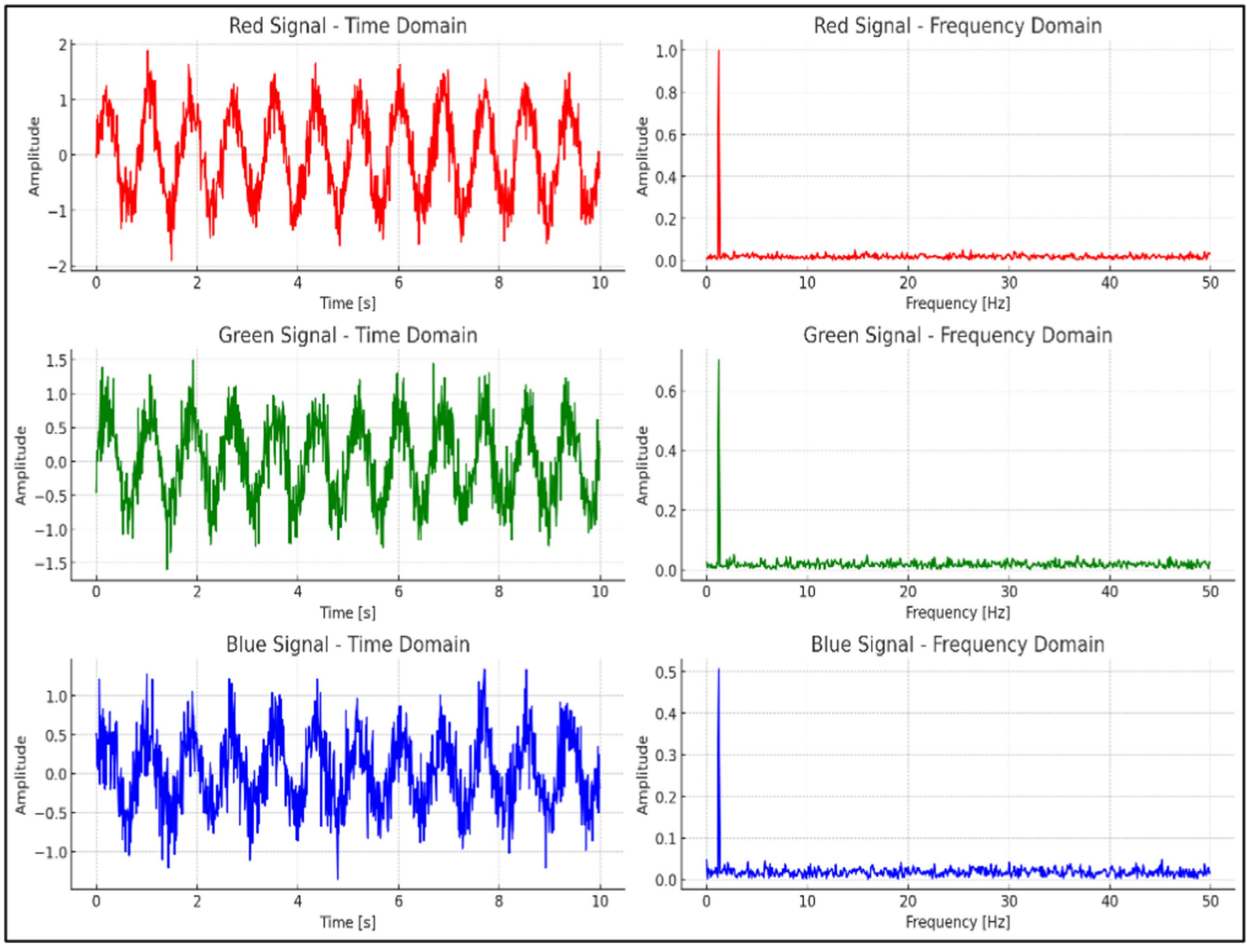
RGB signal analysis in time and frequency domains.

As illustrated in Figure 7, the time-domain and frequency-domain analyses of the RGB signals extracted from the video data are presented. The red channel exhibits the most distinct and consistent periodic waveform in the time domain, while its frequency-domain analysis reveals a sharp, well-defined peak corresponding to the heart rate range. In comparison to the green and blue channels, the red channel demonstrates a higher signal-to-noise ratio (SNR) and more effectively captures the heart rate signal with minimal noise interference. Therefore, the red channel was selected as the optimal channel for heart rate extraction in this experiment.

The next processing step was to average the intensity pixel values over the image sequences of the selected ROI from the R-component of the RGB color space, expressed as Equation (4)


(4) $$i_{R}\left(t\right)=\frac{\sum_{x,y\in ROI}I\left(x,y,t\right)}{\left|ROI\right|}$$

where $I\left(x,y,t\right)$ signifies the intensity pixel value at image location $\left(x,y\right)$ over time $\left(t\right)$ from the recorded frames, and |ROI| refers to the size of the selected ROI. A wavelet signal-denoising method based on an empirical Bayesian method with a Cauchy prior was employed to remove motion artifact noise from $i_{R}\left(t\right)$ , which was induced by pet movement during recording. MATLAB’s built-in command, called “wdenoise,” was used to denoise the signal at four levels through the wavelet Daubechies family (db15), with a universal threshold as a denoising method and a level-dependent approach as a noise estimation method at each resolution level (30). The process of signal denoising was followed by applying a moving average filter with a span equal to 5 to smooth the denoised signal using MATLAB’s built-in command, “smooth.”

#### 2.3.6 Spectral analysis and heart rate extraction

A spectral analysis method based on the FFT was applied to transform the smoothed signal $i_{R}$ smoothed $\left(t\right)$ , from the time domain to the frequency domain. An ideal separating band-pass filter with selected frequencies was then adopted according to the HR range of the pet to separate the HR signal. After passing through the bandpass filter, the vital signs are used as the input signal of the continuous wavelet transformation. An appropriate decomposition level is set to reduce the aliasing phenomenon of the decomposed natural mode components. The value was selected based on actual measured signals.

The decomposed natural mode components are transformed via 1,024-point FFT to obtain the spectrum information. Due to the dogs and cats’ heartbeat frequency ranges (2–4 Hz, respectively), the frequency selector can be used to extract the final HR successively. An inverse FFT was applied to the filtered signals to obtain the HR signals, $I_{R_{H}}\;\left(t\right)$ .

#### 2.3.7 CWT for peak detection and HR estimation

Subsequently, a peak detection method based on the wavelet transform was employed to identify the periodicity of the peaks, their locations, and the number of peaks in the acquired signals. CWT was defined as the scalar multiplication of the acquired signals, $I_{R_{H}}\;\left(t\right)$ , and scaled, shifted versions of the wavelet mother function ψ for each signal.

**Figure 8:**
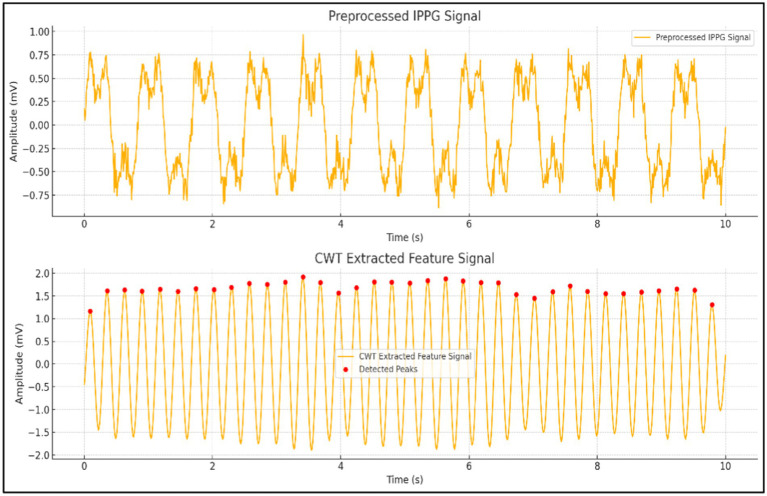
Comparison of preprocessed iPPG signal and CWT-extracted features.

As illustrated in Figure 8, this image presents a comparison between preprocessed iPPG signals and feature signals extracted using CWT. The top plot displays the iPPG signal post-preprocessing, while the bottom plot highlights the smoother and more periodic feature signal derived from CWT, with detected peaks marked. CWT offers significant advantages, including superior time-frequency resolution, multi-scale analysis capabilities, and effective noise suppression, making it an ideal method for accurately extracting features from iPPG signals.

Mathematically, the CWT functions of the signals $I_{R_{H}}\;\left(t\right)$ at points $\left(s,h\right)$ are described as Equation (5) (43, 44).


(5) $$W_{H_{\left(s,h\right)}}=\overset{+\infty }{\underset{-\infty }{\int }}I_{R_{H}}\left(t\right)\psi_{s,h}\left(t\right)dt,\psi_{H_{s,h}}\left(t\right)=\frac{1}{\sqrt{s}}\psi \left(\frac{t-h}{s}\right)$$

where $I_{R_{H}}\;\left(t\right)$ represents the HR signals after denoising and smoothing and $\psi_{H_{s,h}}\;\left(t\right)$ indicates the wavelet function $\psi \left(t\right)$ of the HR signal. Both are translated by the scale s and shifted by h . The outcome of the CWT coefficients contains patterns of peaks and periodicity and can be used to detect the number of peaks, their locations, and their strengths in both signals. All the peaks in $I_{R_{H}}\;\left(t\right)$ , regardless of their width, can be detected because varying scales in wavelet functions yield wavelets with different widths.

Finally, HR (beats per minute b/m) is calculated by Equation (6).


(6) $$HR=\frac{60pF_{r}}{n}$$

where p denotes the number of peaks in the acquired signal, n refers to the number of frames in the selected video, and $F_{r}$ denotes the video frame rate.

### 2.4 Data MATLAB graphical user interface

**Figure 9:**
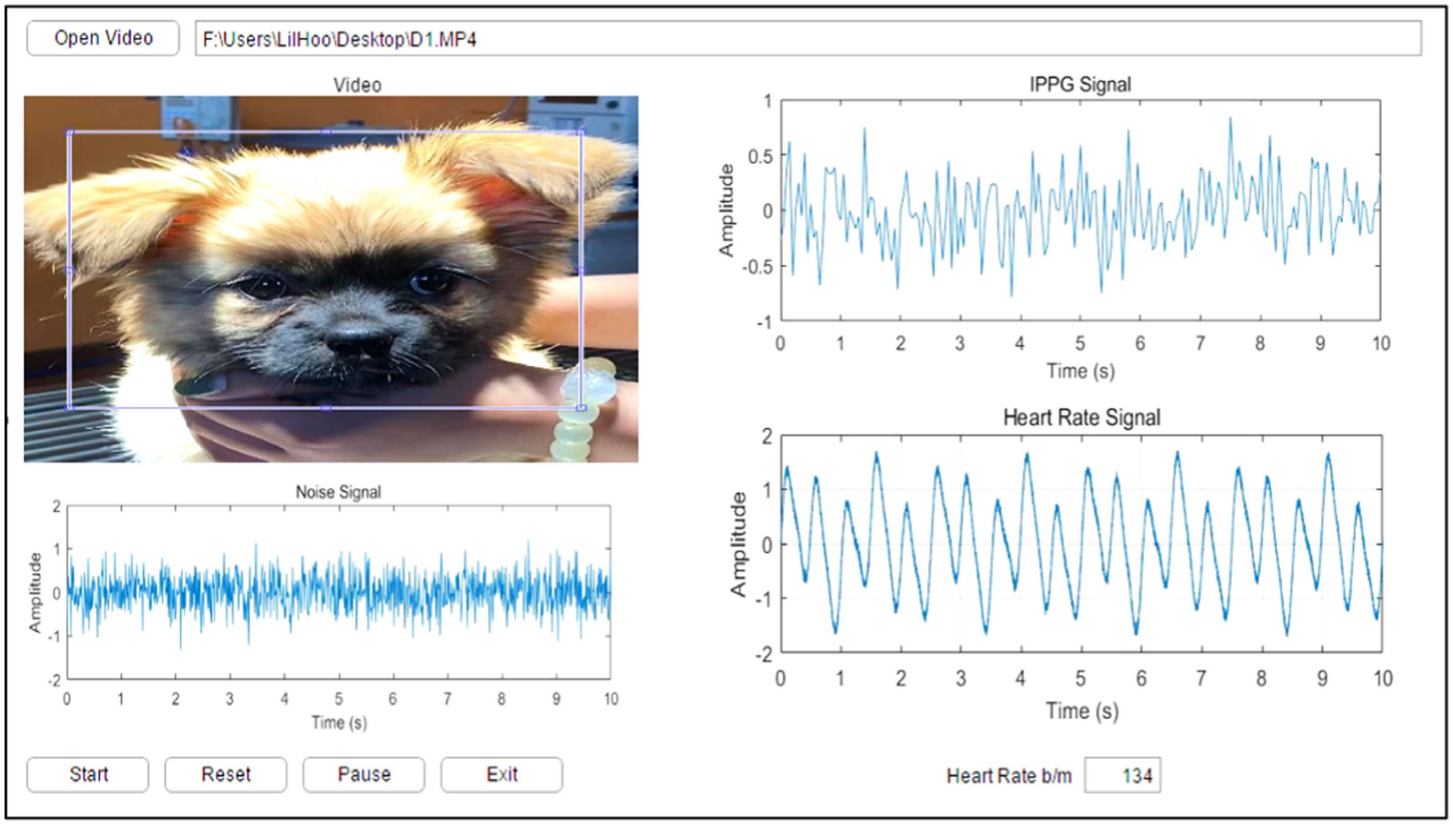
GUI main panel of the proposed image analyzing system.

A GUI model was implemented in MATLAB R2016a (MathWorks, NSW, Australia) with a Microsoft Windows 10 operating system to enable the user to load video data, manually select the cardiopulmonary range for pets, select the ROI where the cardiopulmonary signal was most apparent, and execute the algorithm. The experimentally proposed GUI provides an easy tool to observe video information and the selected ROI and enables the user to recognize the cardiopulmonary readings of the pet. Figure 9 shows the main GUI panel of the proposed experimental image analysis system.

The upper left of the GUI panel displays the input video. Upon clicking the “Start” button, a single frame from the video appears in the top left corner of the panel, where the user can manually define the ROI corresponding to the area with the most visible cardiopulmonary activity. The middle section of the GUI showcases the extracted signals, including the noise signal, the iPPG signal, and the heart rate signal. In the lower part of the GUI, the HR reading for the selected pet is displayed. Additional buttons on the panel allow the user to start, reset, pause, or exit the process as needed.

## 3 Results

This section demonstrates the feasibility of the proposed iPPG for estimating the HR based on the motion of the face surface caused by cardiopulmonary activity without touching the body of the pet. The outcomes of this study concerning the HR were consistent with the respective normal cardiopulmonary ranges of the species. A time period of approximately 30 s was selected from the video data to recover the HR for each animal by averaging the measurements over these periods.

Two different lighting conditions (natural light under natural conditions and artificial light) were compared to set up a control group experiment while capturing videos. Second, various shooting distances may affect our results. Thus, the distances were set to 30, 60, and 90 cm for comparison with the adjusted lighting conditions. The ECG device was simultaneously connected to the pet to obtain findings while capturing the video.

### 3.1 Results of dogs

**Figure 10:**
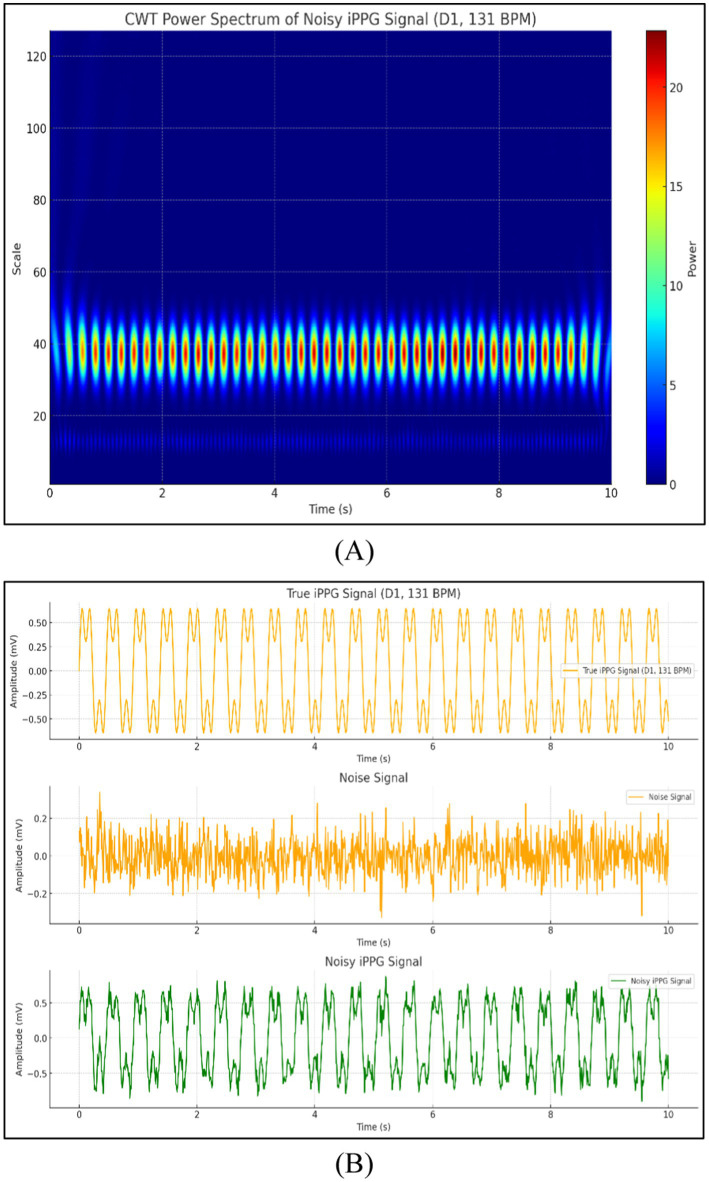
Signals obtained from the video **(A)** CWT power spectrum of noisy iPPG signal and **(B)** comparison of true iPPG, noise, and noisy iPPG signals.

The feasibility of iPPG for estimating HR based on a dog’s face surface is discussed in this section. This experiment was conducted under different settings, and the signals were separated from videos recorded under natural and artificial light conditions. Filters were used to process iPPG signals. Additionally, the extracted iPPG signals were transformed into the average HR estimates to demonstrate the importance of heart rate estimation quality. The signals obtained from the video were processed and analyzed in the form of pictures, as shown in Figure 10.

The data collected from the six dogs were then analyzed statistically to characterize iPPG performance and evaluate whether this kind of system could be used for dog HR monitoring. Data for all dogs were obtained using iPPG.

First, we summarized the average HR of the dogs from the ECG and iPPG and the data, as listed in Table 2. Abs. Error is the absolute error, whereas Rel. Error is the relative error.

**Table 2:** HR average values of dogs detected by ECG and iPPG under the natural light.

Illumination	Subject	ECG	Distance (cm)	iPPG	Abs. error	Rel. error
Natural light	D1	130	30	126	4	3.08%
			60	134	4	3.08%
			90	133	3	2.31%
	D2	131	30	131	0	0.00%
			60	131	0	0.00%
			90	135	4	3.05%
	D3	166	30	162	4	2.41%
			60	164	2	1.20%
			90	160	6	3.61%
	D4	117	30	115	2	1.71%
			60	116	1	0.85%
			90	113	4	3.42%
	D5	188	30	188	0	0.00%
			60	186	2	1.06%
			90	183	5	2.66%
	D6	215	30	212	3	1.40%
			60	219	4	1.86%
			90	210	5	2.33%

Table 2 presents the performance of the algorithm developed for the estimation of the HR in the iPPG of natural light. A comparison of both monitoring techniques showed that the mean absolute error reached 2.94 beats per minute (BPM); therefore, the mean relative error was 1.89%.

**Figure 11:**
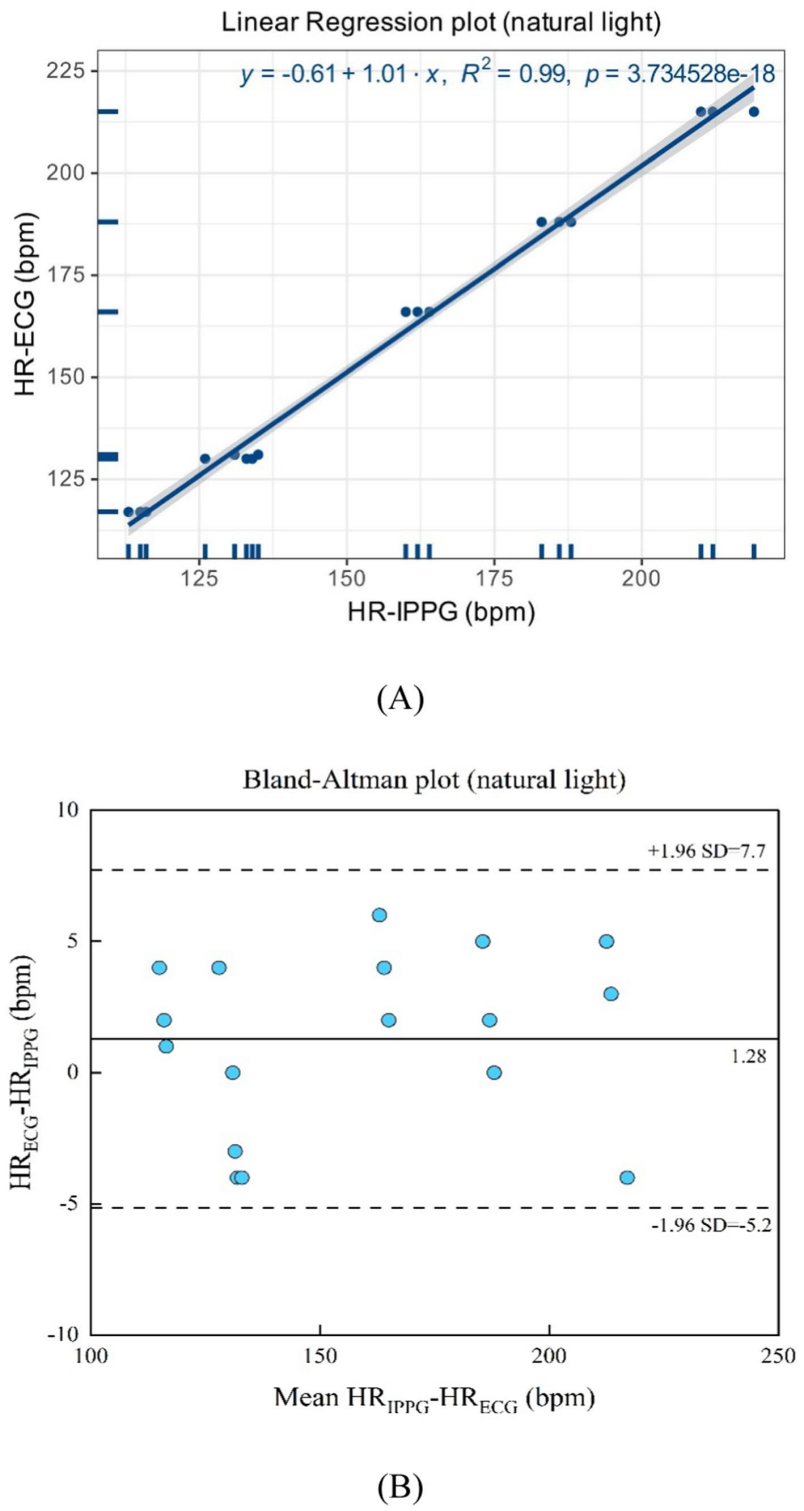
**(A)** Linear regression plot and **(B)** Bland-Altman plot comparing HR assessed with iPPG and HR assessed using ECG under the natural light.

As shown in Figures 11A,B natural light exhibits a linear regression plot and a Bland–Altman plot comparing both monitoring techniques, the iPPG and the HR assessed using an ECG. The results indicated that the *R*-squared (coefficient of determination) was 0.99; the Bland–Altman plot registered a mean difference of 1.28 BPM; the 95% limits of agreement ranged from −5.2 to 7.7 BPM.

The algorithm devised for the estimation of the HR in iPPG under artificial light is presented in Table 3. The comparison of the two monitoring techniques revealed a mean absolute error of 2.94 BPM, resulting in a mean relative error of 1.93%.

**Table 3:** HR average values of dogs detected by ECG and iPPG under the artificial light.

Illumination	Subject	ECG	Distance (cm)	iPPG	Abs. error	Rel. error
Artificial light	D1	130	30	131	1	0.77%
			60	127	3	2.31%
			90	131	1	0.77%
	D2	131	30	131	0	0.00%
			60	134	3	2.29%
			90	135	4	3.05%
	D3	166	30	165	1	0.60%
			60	164	2	1.20%
			90	164	2	1.20%
	D4	117	30	120	3	2.56%
			60	123	4	3.42%
			90	111	6	5.13%
	D5	188	30	182	2	1.06%
			60	193	5	2.66%
			90	185	3	1.60%
	D6	215	30	210	5	2.33%
			60	209	6	2.79%
			90	213	2	0.93%

**Figure 12:**
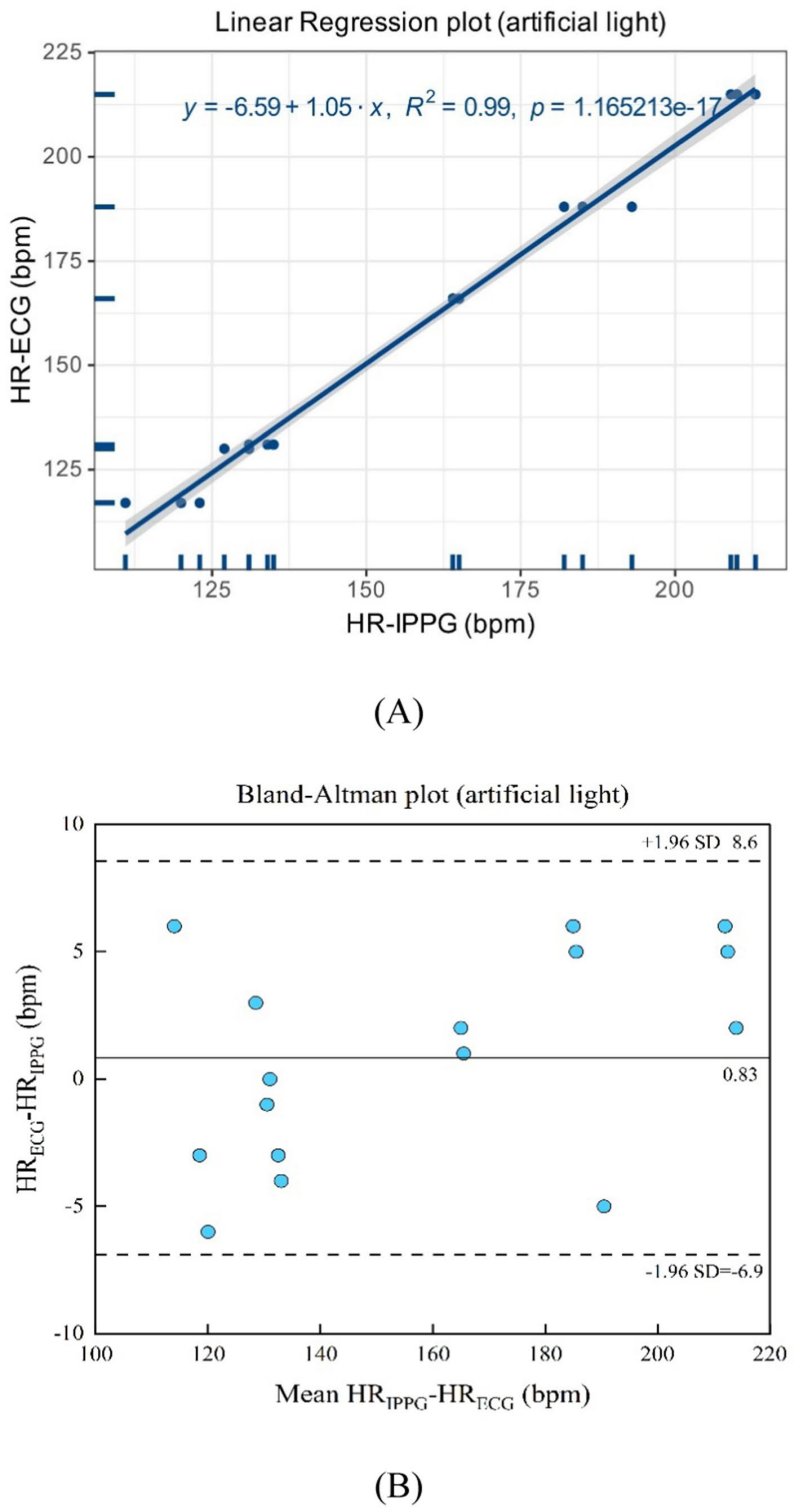
**(A)** Linear regression plot and **(B)** Bland-Altman plot comparing HR assessed with iPPG and HR assessed using ECG under the artificial light.

As suggested in Figures 12A,B, the artificial light presents a linear regression plot and a Bland–Altman plot comparing both monitoring techniques, the iPPG, and the HR assessed using an ECG. The results demonstrated that the *R*-squared (coefficient of determination) was 0.99; the Bland–Altman plot registered a mean difference of 0.83 BPM; the 95% limits of agreement ranged from −6.9 to 8.6 BPM.

**Figure 13:**
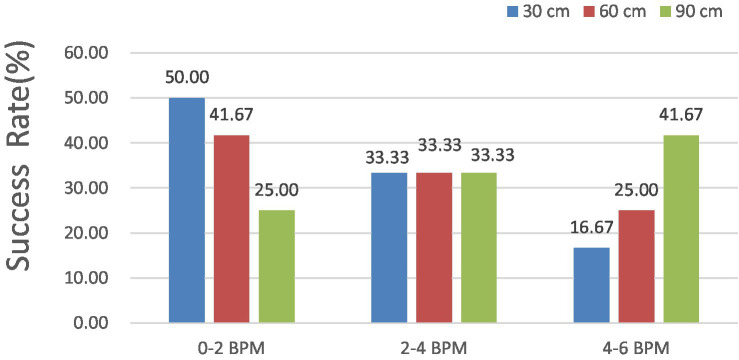
Success HR of the iPPG measurements.

In Figure 13, the HR error is analyzed at shooting distances of 30, 60, and 90 cm. The results indicated that the HR error remained consistently below 6, 4, and 2 BPM for 100, 83.33, and 50% of the measurements, respectively. With respect to the measured external factor of 60 cm, all HR errors remained under 6 BPM for 100%, 4 BPM for 75.00%, and 2 BPM for 41.67% of the measurements.

For the measured external factor of 90 cm, all the HR errors remained under 6 BPM for 100%, 4 BPM for 58.33%, and 2 BPM for 25.00% of the measurements, indicating a better detection success rate.

Notably, the accuracy of the HR measurements obtained through iPPG was excellent, with errors of less than 6 BPM in all test cases, showing that iPPG can correctly estimate the dog’s HR at a typical distance under the configuration involved.

### 3.2 Results of cats

**Figure 14:**
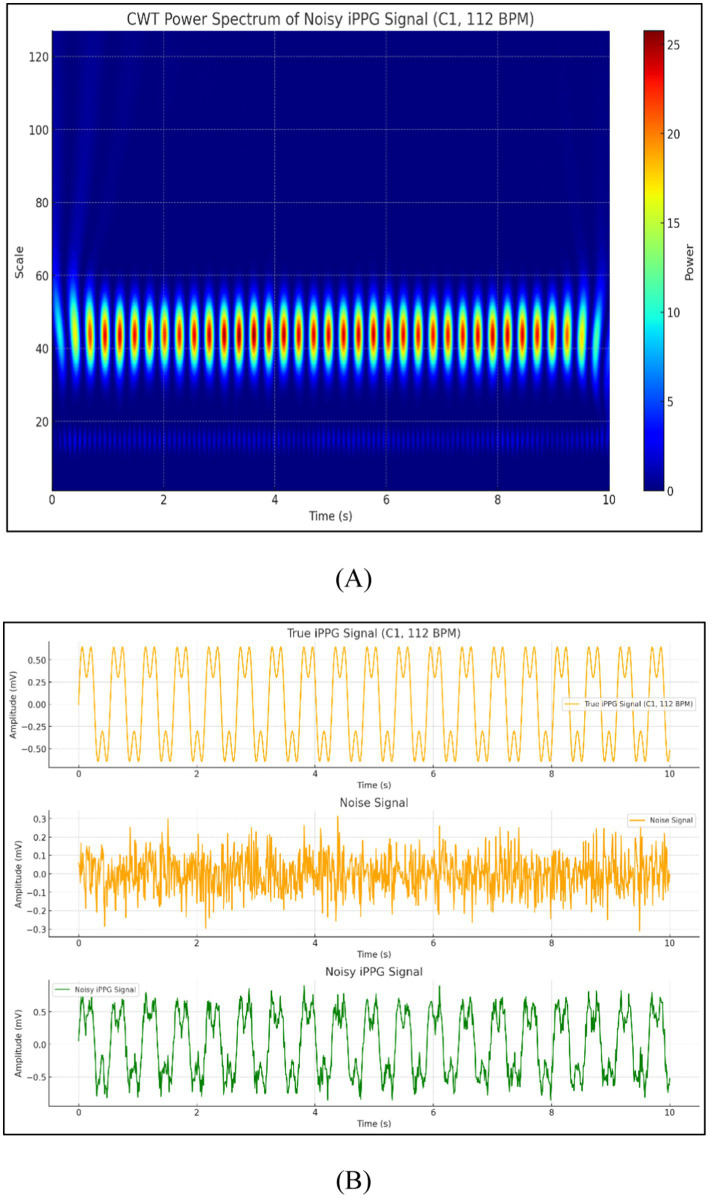
Signals obtained from the video **(A)** CWT power spectrum of noisy iPPG signal and **(B)** comparison of true iPPG, noise, and noisy iPPG Signals.

The feasibility of iPPG for estimating HR based on a cat’s face surface is described in this section. This experiment was conducted under different settings, and the signals were separated from videos recorded under natural and artificial light conditions. In addition, the signals obtained from the video were processed and analyzed in the form of pictures, as shown in Figures 14A,B.

The data collected from the five cats were then statistically analyzed to characterize iPPG performance and evaluate whether this kind of system could be properly used for cat HR monitoring. Data for all cats were measured using iPPG.

First, a summary of the average HR of the cats from the ECG and iPPG was made, and the data of the two heart rates were analyzed, as detailed in Table 4. Table 4 lists the performance of the algorithm developed for the estimation of the HR in the iPPG of natural light. The comparison of the mean absolute error of both monitoring techniques reached 3.33 BPM; therefore, the mean relative error was 2.40%.

**Table 4:** HR average values of cats detected by ECG and iPPG under the natural light.

Illumination	Subject	ECG	Distance (cm)	iPPG	Abs. error	Rel. error
Natural light	C1	115	30	112	3	2.61%
			60	111	4	3.48%
			90	110	5	4.35%
	C2	220	30	216	4	1.82%
			60	218	2	0.91%
			90	214	6	2.73%
	C3	177	30	176	1	0.56%
			60	182	5	2.82%
			90	175	2	1.13%
	C4	94	30	97	3	3.19%
			60	98	4	4.26%
			90	99	5	5.32%
	C5	218	30	217	1	0.46%
			60	220	2	0.92%
			90	221	3	1.38%

**Figure 15:**
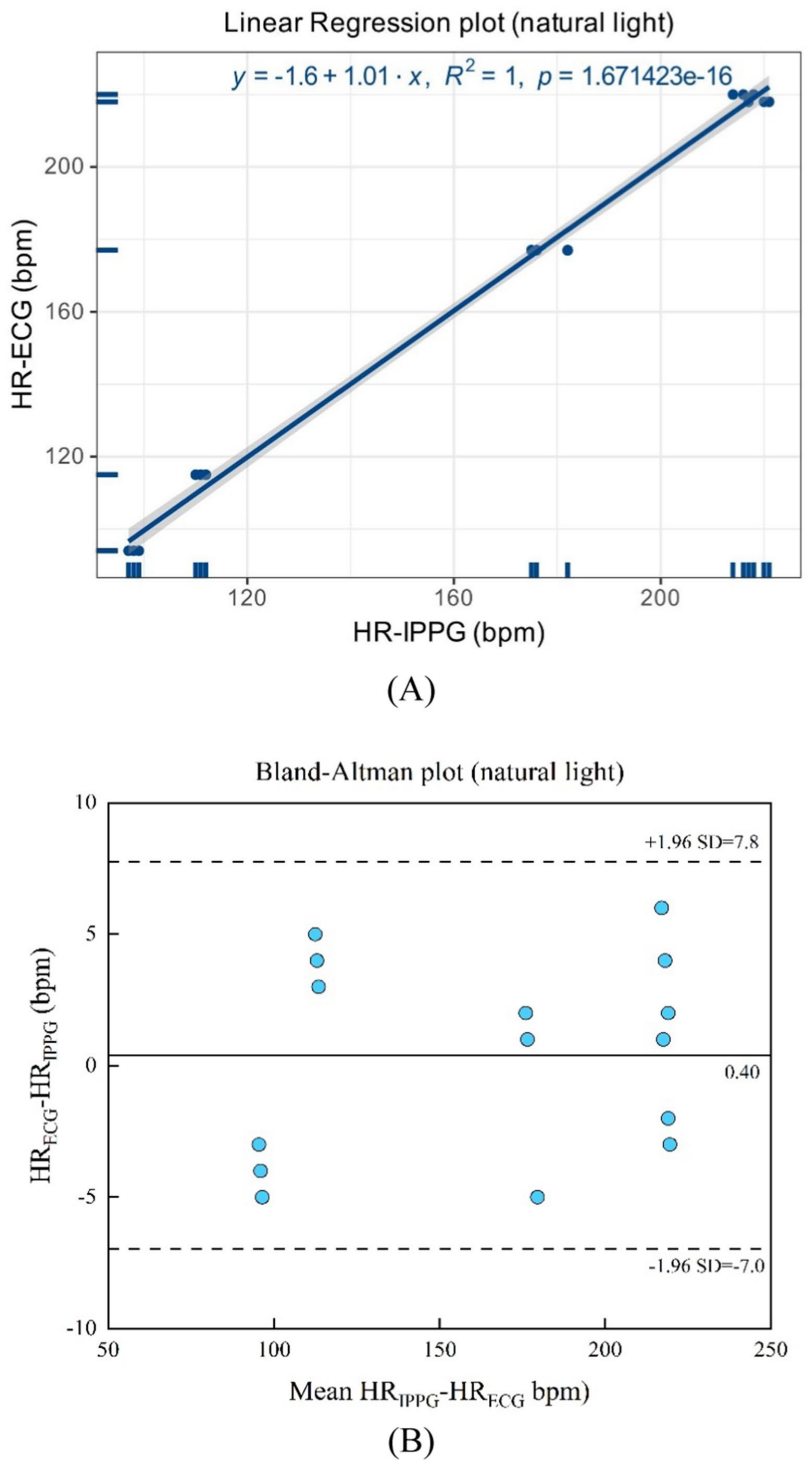
**(A)** Linear regression plot and **(B)** Bland-Altman plot comparing HR assessed with iPPG and HR assessed using ECG under the natural light.

As shown in Figures 15A,B, natural light presents a linear regression plot and a Bland–Altman plot comparing both monitoring techniques, iPPG, and HR assessed using an ECG. The results demonstrated that the *R*-squared (coefficient of determination) was 1; the Bland–Altman plot registered a mean difference of 0.40 BPM; and the 95% limits of agreement ranged from −7.0 to 7.8 BPM.

The performance of the algorithm developed for the estimation of HR in the iPPG of artificial light is presented in Table 5. The comparison of both monitoring techniques of the mean absolute error reached 2.33 BPM; therefore, the mean relative error was 1.65%.

**Table 5:** HR average values of cats detected by ECG and iPPG under the artificial light.

Illumination	Subject	ECG	Distance (cm)	iPPG	Abs. error	Rel. error
Artificial light	C1	115	30	114	1	0.87%
			60	118	3	2.61%
			90	118	3	2.61%
	C2	220	30	222	2	0.91%
			60	221	1	0.45%
			90	220	0	0.00%
	C3	177	30	173	4	2.26%
			60	174	3	1.69%
			90	174	3	1.69%
	C4	94	30	96	2	2.13%
			60	97	3	3.19%
			90	97	3	3.19%
	C5	218	30	214	4	1.83%
			60	221	3	1.38%
			90	218	0	0.00%

**Figure 16:**
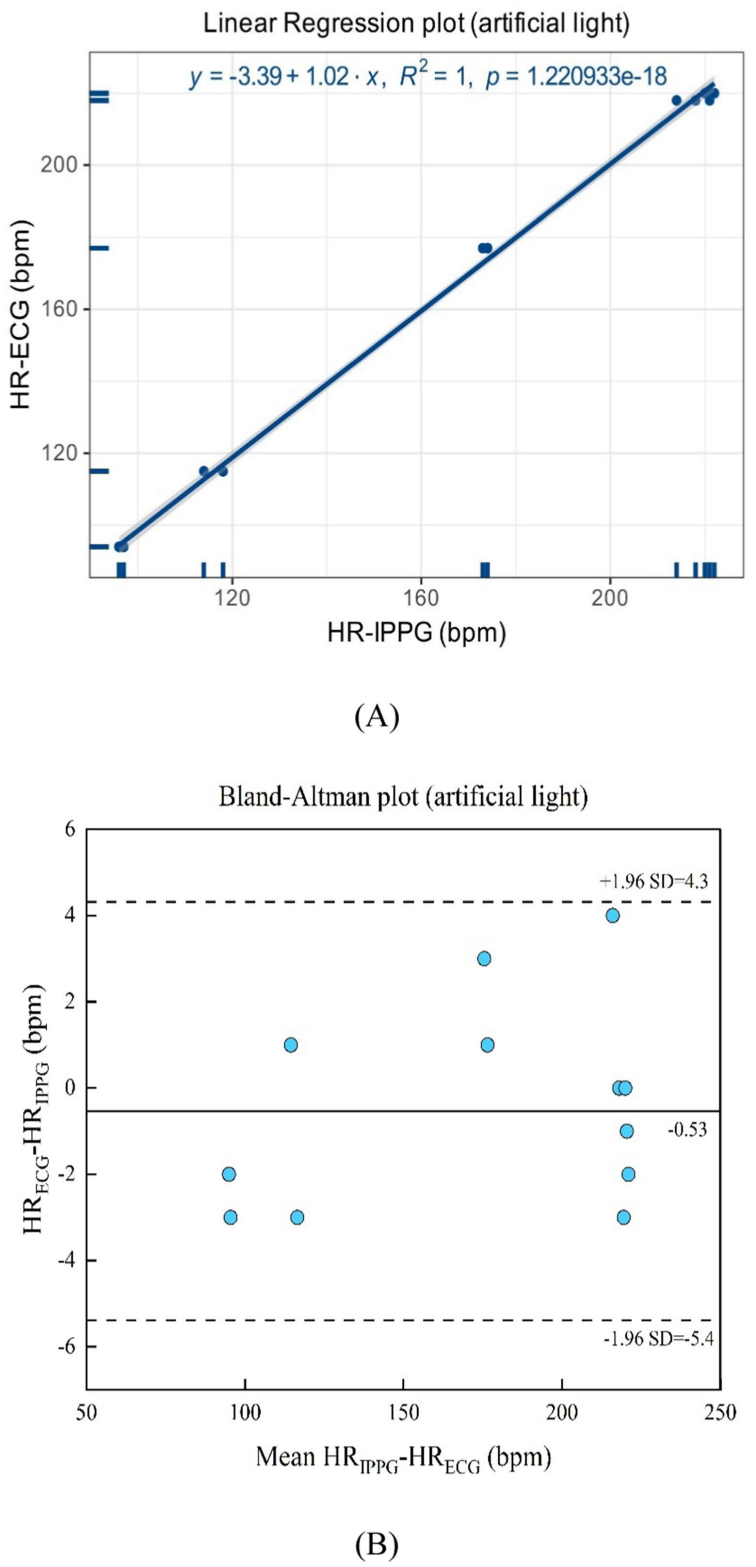
**(A)** Linear regression plot and **(B)** Bland-Altman plot comparing HR assessed with iPPG and HR assessed using ECG under the artificial light.

In Figures 16A,B, the artificial light presents a linear regression plot and a Bland–Altman plot comparing both monitoring techniques, iPPG, and HR assessed using an ECG. The results unveiled that the *R*-squared (coefficient of determination) was 1; the Bland–Altman plot registered a mean difference of 0.53 BPM; the 95% limits of agreement ranged from −5.4 to 4.3 BPM.

**Figure 17:**
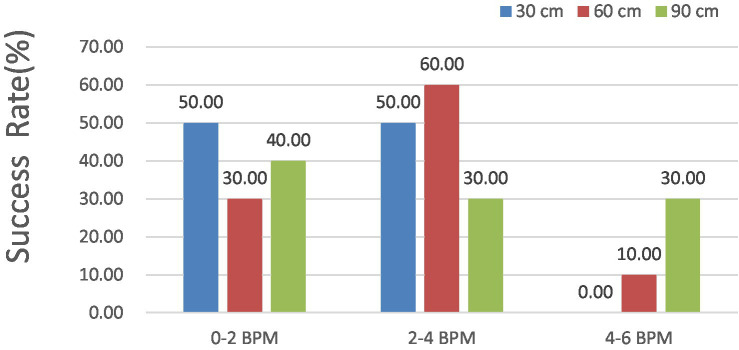
Success HR of the iPPG measurements.

In Figure 17, with shooting distances of 30, 60, and 90 cm, the HR error for 30 cm was maintained below 4 and 2 BPM in 100 and 50% of the measurements, respectively. For a measured external factor of 60 cm, all the HR errors remained under 6 BPM for 100%, 4 BPM for 90%, and 2 BPM for 30% of the measurements. For the measured external factor of 90 cm, all the HR errors remained under 6 BPM for 100%, 4 BPM for 70%, and 2 BPM for 40% of the measurements, suggesting a better detection success rate.

Notably, the accuracy of HR measurements using iPPG was reliable across different shooting distances. Particularly, when the distance was 30 cm, the HR error was always less than 4 BPM in all the cases studied.

Moreover, when the distance was increased to 60 cm, the HR error remained within the acceptable range of less than 90 cm. These results indicate that the iPPG method can accurately estimate the HR of cats at typical distances in the relevant configurations.

## 4 Discussion

This study aimed to monitor the heart rates of cats and dogs using iPPG. Video-based measurement is an unrestricted and non-contact technology that can provide a more comfortable monitoring environment for animals. It also avoids the stress created by the touch sensor, which affects the subject’s physiological parameters. Compared to existing approaches, the proposed method is not influenced by animal hair. Thus, it does not necessitate the removal of the target’s hair before measurement, which is critical for animal welfare (45).

The accuracy of HR measurements for cats and dogs was verified using a contact pressure sensor as a reference. In the experiment, the subjects were awake rather than under anesthesia or recovering from anesthesia. The experimental results demonstrated that iPPG had high measurement accuracy. iPPG, as a contactless measurement device, has no restraint on pets and is less affected by pet hair, making it useful for HR monitoring (46). The study further demonstrates the feasibility of using iPPG for heart rate monitoring in both cats and dogs under varying lighting and distance conditions. Compared with ECG, iPPG showed strong correlation and accuracy, validating its reliability as a non-invasive alternative to traditional methods. One major advantage of iPPG is its ability to preserve animal welfare, as it eliminates physical contact and reduces stress associated with HR monitoring. By using positive reinforcement and familiar environments, animal agitation was minimized, ensuring accurate measurements (47, 48). Additionally, iPPG’s ability to perform without sedation is a key benefit, making it suitable for continuous, long-term monitoring in real-world settings.

Pets tend to move during filming, whereas a human subject may be instructed not to move. Some of these movements might be compensated for using image-processing techniques to extract the different components of movement. These improvements curtailed the variations that were substantially recorded. Considering that the technique is non-invasive, uses less energy, and is unobtrusive, it is possible to obtain even more meaningful data by focusing on long-term observations. This may reveal trends and achieve more stable average readings.

One of the major advantages of iPPG is its noninvasiveness. Unlike traditional methods of HR monitoring, such as ECG, iPPG does not require any physical contact or invasive procedures, making it more comfortable for pets. Furthermore, iPPG can be used remotely, which suggests that it can monitor the HR of a pet even when the pet is moving around or in a natural environment (49, 50).

Limited by experimental equipment, the accuracy of pet HR has not been fully confirmed under surgical anesthesia conditions. Although the pressure sensor could measure the vital signs of pets, the data obtained had limitations. For example, obtaining an accurate HR as the ground truth for iPPG was difficult. Therefore, the results of iPPG measurements of pet HR will be compared with those of ECG data under anesthesia conditions in our follow-up research.

In conclusion, iPPG has potential as a contactless HR monitoring technique in dogs and cats. Its generalizability to experimental and ethology animal facilities can be considered as a significant contribution to animal welfare. Although iPPG has some limitations, further research can help overcome these challenges and make it a valuable tool in pet healthcare.

## 5 Conclusion

As integral members of the modern family unit, pets deserve meticulous attention and care in matters relating to their physical health owing to their limited capacity for communication. This study proposed a novel, non-contact approach for measuring the HR in both feline and canine subjects. This innovative method employs a video-based system that features a user-friendly configuration, delivering previously unattainable levels of precision while enhancing existing animal health monitoring programs without compromising the welfare or circadian rhythms of the subjects in question. The non-invasive design of the system eliminates the risk of harm or discomfort to the animals while simultaneously affording caregivers invaluable data to support informed decisions concerning the well-being of their pets.

In summary, the non-invasive video-based HR extraction method proposed in this study can be generalized to experimental situations that have not been addressed in previous studies. To the best of our knowledge, the iPPG method successfully extracted HR estimates for pets from RGB videos, was significantly robust to small head movements, and successfully tracked HR in awake cats and dogs. Thus, it is a noninvasive, low-cost, and easy-to-implement HR tracking method that can be used in multiple animal behavioral, veterinary, and experimental setups. Moreover, association with animals will be further considered to generalize this method to open-field situations, contributing to a real breakthrough in the study of animal behavior and well-being.

## Data Availability

The original contributions presented in the study are included in the article/supplementary material, further inquiries can be directed to the corresponding author.

## Footnotes

^1^ http://opencv.org

^2^ www.mathworks.com
